# Duplication of *SOX3* in an *SRY*-negative 46,XX male with prostatic utricle: case report and literature review

**DOI:** 10.1186/s12920-022-01347-0

**Published:** 2022-09-05

**Authors:** Jiansheng Wei, Changrong Liu, Minyan Zhang, Shen Liu, Junjie Fu, Peng Lin

**Affiliations:** Department of Pediatric Urology, Fuzhou Children’s Hospital of Fujian Province, 145 Ba-yi-qi road, Fuzhou, Fujian Province China

**Keywords:** Disorder of sex development, *SOX3*, Prostatic utricle, Case report

## Abstract

**Background:**

46,XX male disorders of sex development are rare. Approximately 80% of cases of testicular tissue differentiation may be due to translocation of *SRY* to the X chromosome or an autosome. *SRY*-negative 46,XX males show overexpression of pro-testis genes, such as *SOX9* and *SOX3,* or failure of pro-ovarian genes, such as *WNT4* and *RSPO1*, which induces testis differentiation, however, almost all testicles exhibit dysgenesis. Following inadequate exposure to androgens during the embryo stage, remnants of the Mullerian duct and incomplete closure of the urogenital sinus lead to enlargement of prostatic utricles. This condition is associated with proximal hypospadias and disorders of sex development. Many cases are asymptomatic, but show increased rates of postoperative complications and surgical failure.

**Case presentation:**

A 5-year-old Chinese boy with scrotal hypospadias and bilateral cryptorchidism with prostatic utricles was presented. Gonadal histology showed ovo-testicular tissue on the right side and testicular tissue on the left side; all testicular tissue exhibited dysgenesis. Furthermore, chromosome karyotype analysis revealed 46,XX and, the presence of SRY was ruled out by polymerase chain reaction analysis. Whole-genome analysis showed the boy has a 1.4-Mb duplication in the Xq27.1q27.2 region (arr[hg19]Xq27.1q27.2:139585794–140996652) involving SOX3. No SOX3 duplication was observed in the parents, who had a normal phenotype.

**Conclusions:**

We report the first case of an SRY-negative 46 XX male with prostatic utricle caused by SOX3 duplication. *SOX3* duplication may cause sex reversal, and all 46,XX *SRY*-negative males should be screened for *SOX3* mutations. Gonadal biopsy is recommended to evaluate ovarian and testicular tissue development. Testicular dysgenesis and low exposure to male hormones during fetal development can lead to enlarged prostatic utricles. Thus endoscopic examination should be performed preoperatively to detect prostatic utricles in *SRY*-negative 46,XX males to determine the surgical plan and reduce postoperative complications.

## Background

Ovotesticular male disorders of sex development (OT-DSD) are rare worldwide. These disorders show differing prevalence and karyotypes; however, the 46,XX karyotype is the most common(65–90%) [1]. This presentation results from the translocation of *SRY* to the X chromosome or, more rarely, to the autosomes. The *SRY*-negative phenotype is rare and can be explained by two different mechanisms: overexpression of pro-testis genes, such as *SOX9* and *SOX3,* and failure of pro-ovarian genes, such as *WNT4* and *RSPO1* [2]. *SOX3* mutation-induced sex reversal may be driven by the shared analogous function of *SOX3* and *SRY*, cooperation with SF1 to upregulate *SOX9* expression, and induction of Sertoli cell differentiation in the bipotential gonad ridges [2]. A previous study reported cases of hypospadias, bilateral cryptorchidism, and kidney abnormality [3]. In our case, we additionally observed an enlarged prostatic utricle, which to our knowledge is the first instance of this phenotype observed in this specific type of DSD. The embryological origin of the prostatic utricle remains controversial. It was previously considered as a remnant of the Müllerian ducts, however, a recent study showed it to be derived from the urogenital sinus [4]. Either way, the prostatic utricle dilates when exposure to male hormones is low during fetal development [5]. In an *SRY*-negative 46,XX male, lack of the Y chromosome results in inadequate differentiation of the testis, an insufficient hormone dosage during critical periods, induction of remnant Mullerian ducts and incomplete closure of the urogenital sinus [6].

## Case presentation

**Fig. 1:**
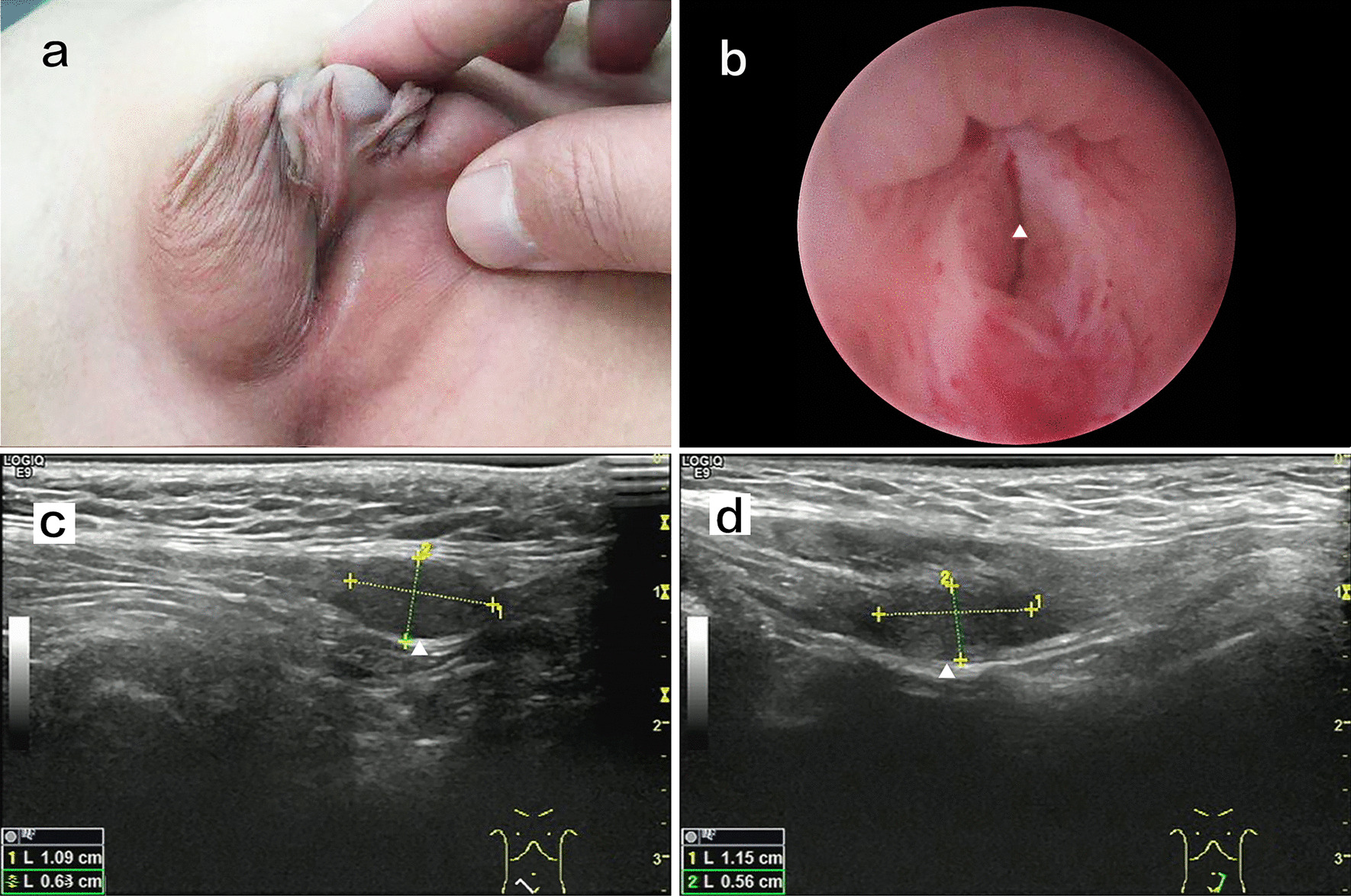
**a** Physical examination revealed scrotal hypospadias and bilateral cryptorchidism. **b** Pre─operation cystoscopy showing an orifice in the prostatic urethra; the white arrow indicates the prostatic urethra orifice. **c** Ultrasonography showing that the right testicle is in the inguinal canal; the morphology was normal. **d** Ultrasonography showing that the left testicle is in the inguinal canal; the morphology was normal

The patient was 5 years old and was referred to our department for ambiguous sex; he was the second child of a non-consanguineous couple. His parents and sister were healthy. The patient’s birth weight was 3.5 kg and length was 50 cm, which were within normal ranges; his intelligence was normal, and he had no pertinent medical history. At 5 years old, the patient’s height was 117 cm and weight was 20.5 kg. Physical examination revealed scrotal hypospadias and bilateral cryptorchidism (Fig. 1a). The bilateral gonads (approximately 1.0 × 0.6 cm) were palpated in the upper inguinal region. Ultrasonography indicated bilateral cryptorchidism with a normal testicular morphology (Fig. 1c, d). Abdominal ultrasound did not show internal female genitalia, and echocardiogram results were normal. Hormonal laboratory tests showed low basal levels of testosterone (T; < 2.5 ng/dL); the post-3-day human chorionic gonadotropin stimulation test showed an increase in T to 62.90 ng/dL, and the post-human chorionic gonadotropin test showed a normal level of double hydrogen testosterone (80.37 pg/mL). Thus, there was a good T response to human chorionic gonadotropin stimulation. Luteinizing hormone was low (< 0.1 IU/L) and follicle stimulating hormone was normal (0.4 IU/L). Anti-Mullerian hormone (48.48 ng/mL), prolactin (4.87 ng/mL), and 17-α-hydroxyprogesterone (0.8 nmol/L)levels were also normal (Table 1). As patient growth and development were normal, we did not screen for growth hormone or adreno-cortico-tropic hormone.

**Table 1:** Hormonal laboratory tests

Hormonal laboratory tests	Result	Reference range
testosterone		
basal	< 2.5 ng/dl	
Post HCG	62.90 ng/dl	
		T difference value > 10 ng/dl
Post HCG DHT	80.37 pg/ml	
		T/DHT < 10
AMH	48.48 ng/ml	Male(> 4 years):37.88–298.52 ng/ml Female(> 4yeays):0.05–7.02 ng/ml
LH	< 0.1 IU/L	0- 4.10 IU/L
FSH	0.4 IU/L	0–1.90 IU/L
Prolactin	4.87 ng/ml	2.00–43.00 ng/ml
17-α-hydroxyprogesterone	0.8 nmol/l	0–11.5 nmol/l

HCG: Human Chorionic Gonadotropin. DHT: Double Hydrogen Testosterone. AMH: Anti-Mullerian hormone. LH: Luteinizing Hormone. FSH: Follicle Stimulating Hormone

**Fig. 2:**
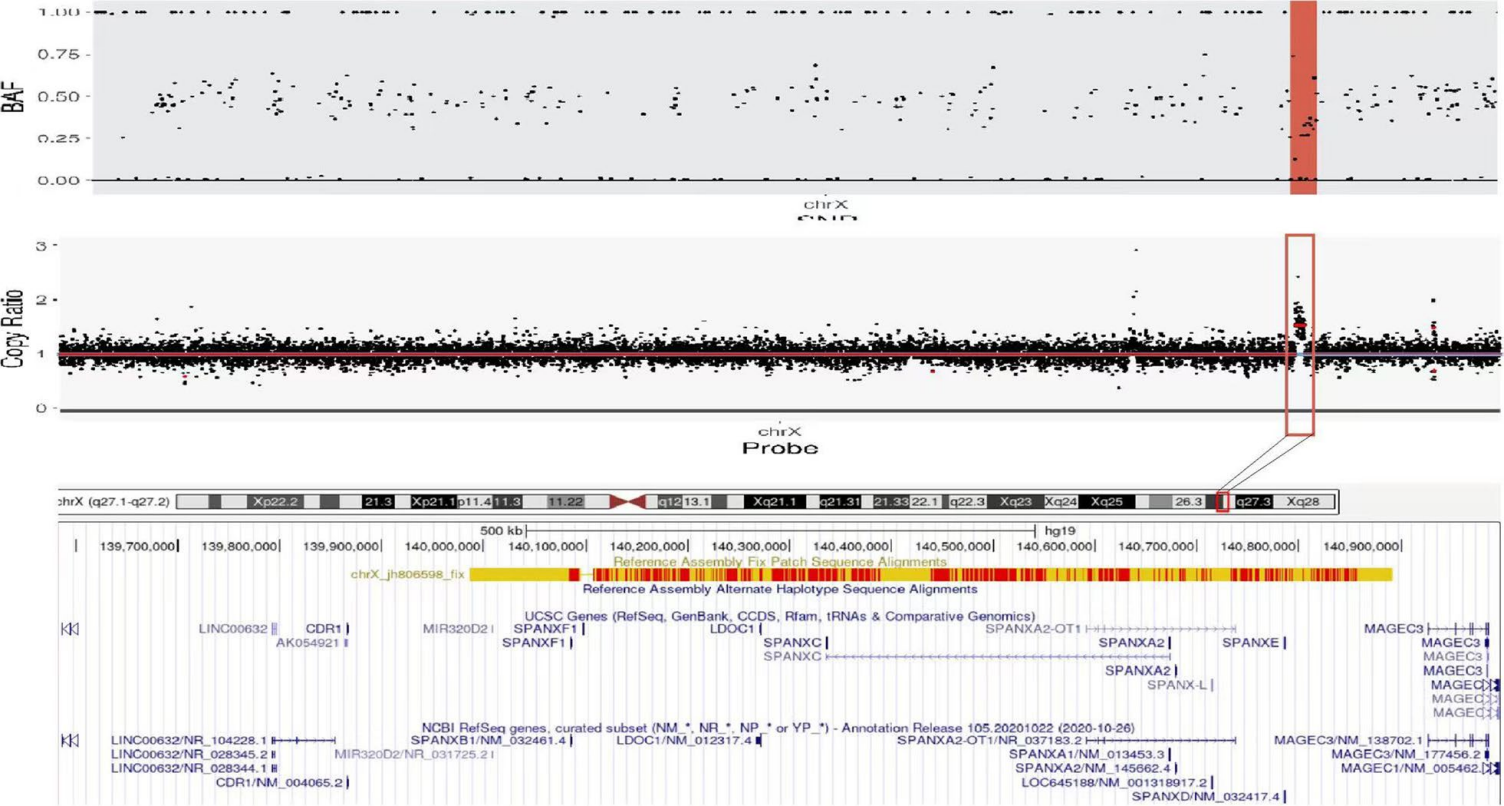
Whole genome analysis showing 1.4-Mb duplication in the Xq27.1q27.2 region (arr[hg19]Xq27.1q27.2:139,585,794–140,996,652). The duplication contained *CDR1*, *SOX3*, and *SPANXA1*

The chromosome karyotype was 46,XX, and the presence of SRY was ruled out by polymerase chain reaction analysis. Therefore, we performed whole-genome analysis for genetic detection. Genomic DNA was extracted from blood samples using the DNeasy Blood & Tissue Kit (Qiagen, Hilden, Germany). The DNA quantity of sequencing library was assessed by Qubit 2.0 fluorometer (Thermo Fisher Scientific, Massachusetts, United States). The quality and size of libraries were measured by 2100 Bioanalyzer High Sensitivity DNA Assay (Agilent Technologies, California, United States). The qualified libraries were sequenced using the 2 × 150-bp paired-end sequencing on the Illumina NovaSeq platform (Illumina, San Diego, USA). FASTQ files were aligned to the human reference genome (hg19/ GRCh37) using BWA v0.7.13 Variants (single nucleotide variants and indels) were genotyped from recalibrated BAM files using GATK 4.0 and annotated against multiple databases, including HGVS variant description, population frequency, disease or phenotype and variant functional prediction, using ANNOVAR. Variants were classified as pathogenic, likely pathogenic, variant of unknown significance, likely benign, or benign following the American College of Medical Genetics (ACMG) guidelines. Copy number variants were called by DNAcopy R package, filtered classified as per the ACMG guidelines, and manually checked by using the Integrative Genomics Viewer. Target capture area was 50 M, detection range capture rate 99.9%, detection data volume 10G, average sequencing depth ≥ 100X, and the proportion of Q30 was not less than 90%. The results revealed no pathogenic or likely pathogenic single-nucleotide variation or insertion-deletion variant related to sexual development, but revealed a 1.4-Mb duplication in the Xq27.1q27.2 region (arr[hg19]Xq27.1q27.2:139,585,794–140,996,652). The duplication contained *CDR1*, *SOX3*, and *SPANXA1* (Fig. 2). According to the ACMG, this duplication is of uncertain significance. The parents did not show *SOX3* duplication and had normal phenotypes.

**Fig. 3:**
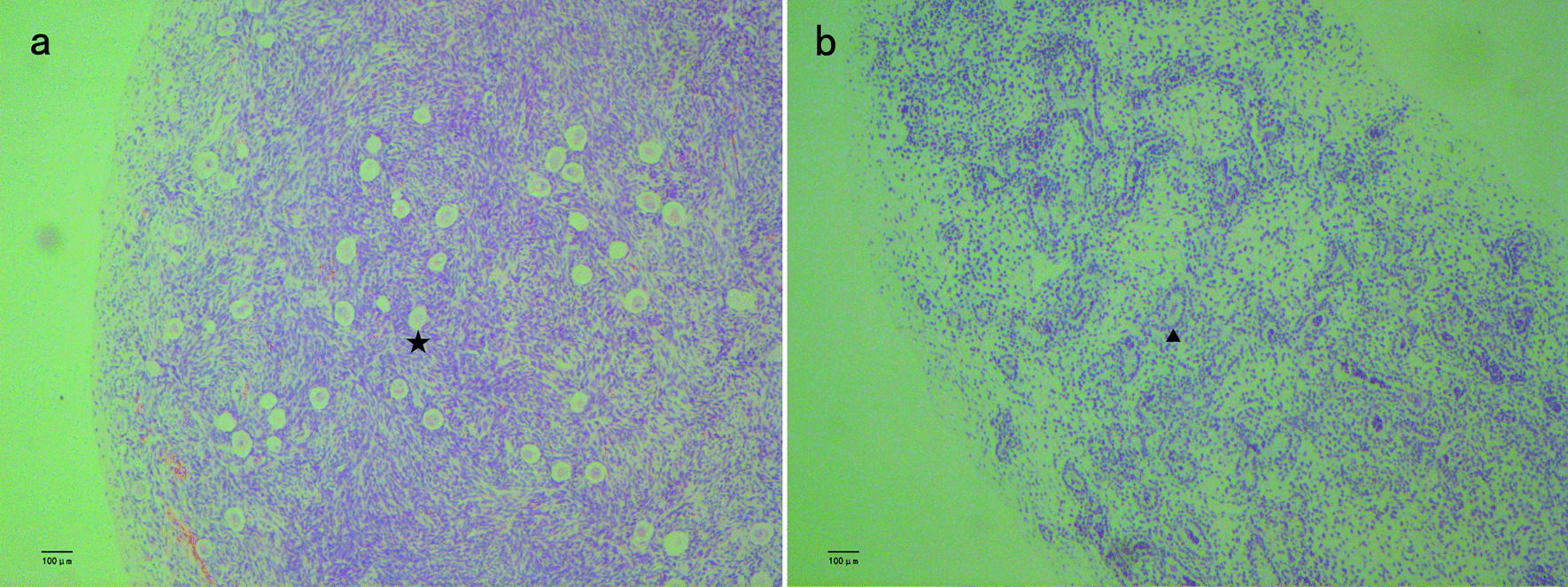
Hematoxylin and eosin staining of bilateral gonadal sample. **a**: The right side showing ovotesticular tissue; the ovarian tissue had primordial and primary follicle(star). **b**: The left side showing testicular dysgenesis(arrow). These images were obtained using the following equipment: microscope DM300 and camera DFC45O(leica, Germany). Scanner Hamamatsu, Nanozoomer S210-NDP. View 2 version 2.9.29, was used as acquisition software and the measurement resolution was 1200dpi

After clinical consultation, the family decided to raise the child as a male. The patient underwent surgical procedures for the correction of hypospadias and gonad biopsy, followed by orchiopexy. Preoperative cystoscopy showed an orifice in the prostatic urethra. Upon probing with a camera, enlarged prostatic utricles were observed (Fig. 1b). The bilateral gonads were biopsied, followed by orchiopexy. Gonadal histology showed ovotesticular tissue on the right side and testicular tissue on the left side; both testicular tissues exhibited features of testicular dysgenesis. The ovarian tissue had primordial and primary follicles (Fig. 3a, b). Owing to this complicated condition, we performed penile straightening and planned to perform urethroplasty 6 months later. After degloving of the penis, the corpus cavernosum was well developed and the penis length and diameter were 4.5 and 1.5 cm, respectively.

## Discussion and conclusion

*SOX3* is a single exon gene located in Xq27.1 and is required for normal brain, pituitary and craniofacial development in humans. Recent reports showed that mutation of *SOX3* can cause developmental delay or intellectual disturbance [7, 8]. Although *SOX3* is not required for normal sex development, it affects testis differentiation and oocyte development [9], Mutation of *SOX3* induces *SRY*- negative 46,XX male sex reversal has been comfirmed by previous reports [10]. However, the mechanism underlying *SOX3* mutation inducing *SRY*-negative 46 XX male sex reversal was not well understood. Through a literature review, we found 10 similar cases (Table 2) [2, 3, 10–15]. Seven of these cases contained *SOX3* duplication mutations, ranging from 550 kb to 6 Mb. The mechanism may involve overexpression of *SOX3* to upregulate SOX9 expression and induce testis differentiation [1]. Our patient had a 1.4-Mb duplication in the Xq27.1q27.2 region, which contained *SOX3*, supporting this prediction. In three of the cases, the condition was associated with rearrangements in the regulatory region of *SOX3*, which may weaken the inhibition of *SOX3* [15].

**Table 2:** Cases of SOX3 related XX male reversal

	Suttou et. al Patients A	Suttou et. al Patients B	Suttou et. al Patients C	4: Moalen et. al	5: Haines.et al	6: Vetro.et	7: Grisponetal.et	8: Tasic.et	9: Zhuang.J et	10: Qin,S et	Our patient
Disorders of sex developmen	XX male reversal	XX male reversal	XX male reversal	XX male reversal	OT-DSD	XX male reversal	OT-DSD	XX male reversal	OT-DSD	XX male reversal	OT-DSD
Age	30 years	Endocrine:19 years histology:26 years	1.5 years	1 years	1.5 months	8 years	2.5 years	11 years	7 years	31 years	5 years
Growth and developmental issues	Normal	Developmental delay;microcephaly	Developmental and growth delay,microcephaly	Normal	normal	Mild intellectual disability	Normal	Normal	Normal	Normal	Normal
Hormone analysis	FSH:22.0mIU/ml LH:11.IIU/L Prolactin 17.9ug/l Free T:1.92 ng/dl	FSH:69.0 mIU/ml LH:35.0 IU/L Prolactin 3.0ug/l Free T:0.86 ng/dl	Unknow	FSH/LH/T:normal	T:9.2 nmol/L Post-HCG:T:2.1 → 24.6 nmol/l DHT:2.0 → 7.5 nmol/l Androstenedione0.6 → 1.1 nmol/l	Unknow	T(nmol/l) Basal: < 0.35 Post-HCG:1.05 AMH:216 LH: < 0.1 FSH0.73	Post HCG: T:146 ng/ml A:T: < 1 T:DHT was 5.6	LH < 0.2mIU/ml FSH:1.21 mIU/ml T: < 0.1 ng/ml Serum progesterone/prolactin:normal	T:1.75 ng/ml Progesterone:0.11 ng/ml Prolactin:208.44uIU/ml LH:29.32mIU/ml FSH:37.88mIU/ml	LH0.4 IU/L FSH0.1 IU/L Prolactin:4.87 ng/ml T: < 2.5 ng/dl AMH:48.48 ng/ml Post-HCG:T:62.9 ng/dl
Genitals	Unkown	penile development with small testis.Shaft length, 10.2 cm; shaft diameter, 2.6 cm	Right testicles appear smaller than left;Hypoplastic scrotum;testes are retractile and can be brought down	Left cryptorchidism	Bifid scrotum; small phallus; distal hypopadias	Normal	Hypospadias and bilateral cryptorchidism	Moderate coronal hypospadias	Hypospadias and bilateral cryptorchidism	Normal	Scrotal hypospadias and bilateral cryptorchidism
Gonadal Histology	No details	Testicular dysgenesis	No details	No details	Right:ovotesticular	No details	The testicular tissue and ovarian tissue all exist.Andtesticular dysgenesis	No details	The ovotesticular tissue on the left side and the testicular tissue on the right side	No details	Right:ovotesticular tissue; Left: testicular tissue Testicular dysgenesis
Associated anomalies	–	–	–	Normal	Fallopian tube, hemiuterus and hemivagina on the right gonads	–	–	Kidney hypodysplasia	–	–	Prostatic utricle
Genotypes	SRY negative Two microduplications were observed,the first of which spanned the entire sox3 gene	Single 343-kb microdeletion on the X-chromosome immediately upstream of SOX3	SRY negative 6 Mb duplication that encompasses sox3 and at least 18 additional distally located genes	SRY negative 0.494 Mb copy number gain in region Xq27.1 which contains the SOX3, RP1-177G6.2, CDR1	SRY negative carries 774 kb insertion translocation from chromosome 1 into a 82 kb distal to SOX3	A 5.6 Mb duplication of the long arm of a chromosome X, involving the SOX3 gene	SRY negative, at Xq27.1. The duplicated region was around 0.5 Mb, and encompassed the SOX3	A unique 550 kb duplication involving SOX3	SRY negative 2.2 Mb duplication that encompasses SOX3 gene	SRY negative, 867 kb heterozygous deletion in Xq27.1,located at 104 kb downstream of SOX3	SRY negative 1.4 Mb duplication that encompasses SOX3,CDR1,SPANXA1

Our patient’s bilateral gonads were biopsied, and histological analysis showed that the testicular component was dysgenetic and that ovarian tissue was present, Therefore, the diagnosis was revised to OT-DSD. Haines et al. [12], Grisponetal et al. [10], and Zhuang et al. [14] also performed gonadal biopsy and obtained similar results. The development of testicular tissue and ovarian tissue may influence the sex assignment of patients with DSD. If the patients are raised as males, then the ovarian portion must be removed before pubertal age to avoid gynecomastia and cyclic hemorrhage. If the patients are raised as females, testicular tissue must be removed to avoid virilization during puberty [1]. Therefore, at any age, when the patient is diagnosed with an *SRY*-negative 46 XX karyotype, the gonads must be biopsied.

Our case involved duplication of *SOX3* in an *SRY*-negative 46,XX male associated with an enlarged prostatic utricle, which has not been reported previously. Tasicet et al. [3] reported patients with congenital anomalies of the kidneys. Haineset al [12] reported patients with fallopian tubes, hemiuterus, and hemivagina on the right gonads, which were removed through surgery. Such patients must also be screened for abnormalities in other organs. Our patient also underwent an ultrasound, but the results were normal. We routinely perform cystoscopic pre-operation in patients with proximal hypospadias, which revealed an enlarged prostatic utricle in the current patient. Ultrasonography and retrograde urethrography can detect the prostatic utricle, but it is difficult to detect a small prostatic utricle using ultrasonography, and retrograde urethrography is not useful because of the slit-like utricular opening [16]. Thus, endoscopy is suitable for detecting the prostatic utricle. An enlarged prostatic utricle can cause recurrent urinary tract infection, epididymo-orchitis, fistulas after urethroplasty, and calculi or malignancy [17]. Early diagnosis is important for providing support for individualized plans and reducing postoperative complications [18]. Therefore, endoscopic examination should be performed in *SRY*-negative 46,XX males with hypospadias, as recommended previously [6].

Our patient has a 1.4-Mb duplication in the Xq27.1q27.2 region. The duplication contained *CDR1*, *SOX3*, and *SPANXA1*. Cerebellar degeneration-related antigen 1(*CDR1*) is expressed in the nervous system, human epidermis, and cancer tissues, but its function is unclear [19]. *SPANXA1* is expressed in the tumors or testis [20], but has not been reported to be associated with sexual reversal. Therefore, the relationship between these two genes and sex determination requires further analysis.

*SOX3* duplication may causes sex reversal, and all 46,XX *SRY*-negative males should be screened for *SOX3* mutations. Gonadal biopsy is recommended to determine the presence of ovarian and testicular tissue development. Testicular dysgenesis and low exposure to male hormones during fetal development can lead to enlarged prostatic utricles. Thus, endoscopic examination should be performed preoperatively to detect prostatic utricles in *SRY*-negative 46,XX males to guide the surgical plan and reduce postoperative complications.

## Data Availability

The datasets (whole-genome analysis, polymerase chain reaction results, hormonal laboratory tests) used and/ or analyzed during the current study are available from the corresponding author on reasonable request. The sequencing data has been deposited in NCBI Bioproject database, The data is accessible via the accession number: PRJNA864577 (https://www.ncbi.nlm.nih.gov/sra/PRJNA864577). The following databases are used in this study: Human Reference Genome (hg19/ GRCh37) (https://www.ncbi.nlm.nih.gov/projects/genome/guide/human/index.shtml) GATK (https://www.broadinstitute.org/gatk/) ANNOVAR(https://annovar.openbioinformatics.org/en/latest/).

## Abbreviations

DSD: Disorders of sex development
OT-DSD: Ovotesticular male disorders of sex development
T: Testosterone
